# Antigen-presenting aged neutrophils induce CD4^+^ T cells to exacerbate inflammation in sepsis

**DOI:** 10.1172/JCI164585

**Published:** 2023-07-17

**Authors:** Hui Jin, Monowar Aziz, Atsushi Murao, Molly Kobritz, Andrew J. Shih, Robert P. Adelson, Max Brenner, Ping Wang

**Affiliations:** 1 Center for Immunology and Inflammation, Feinstein Institutes for Medical Research, Manhasset, New York, USA.; 2 Department of Molecular Medicine and; 3 Department of Surgery, Zucker School of Medicine at Hofstra/Northwell, Manhasset, New York, USA.; 4 Robert S. Boas Center for Genomics and Human Genetics, Feinstein Institutes for Medical Research, Manhasset, New York, USA.

**Keywords:** Cell Biology, Immunology, Neutrophils, Th1 response

## Abstract

Extracellular cold-inducible RNA-binding protein (eCIRP) is a key mediator of severity and mortality in sepsis. We found that stimulation of mouse bone marrow–derived neutrophils (BMDNs) with eCIRP generated a distinct neutrophil subpopulation, characterized by cell surface markers of both antigen-presenting cells and aged neutrophils as well as expression of IL-12, which we named antigen-presenting aged neutrophils (APANs). The frequency of APANs was significantly increased in the blood, spleen, and lungs of WT mice subjected to cecal ligation and puncture–induced sepsis but not in CIRP^–/–^ mice. Patients with sepsis had a significant increase in circulating APAN counts compared with healthy individuals. Compared with non–APAN-transfered mice, APAN-transferred septic mice had increased serum levels of injury and inflammatory markers, exacerbated acute lung injury (ALI), and worsened survival. APANs and CD4^+^ T cells colocalized in the spleen, suggesting an immune interaction between these cells. APANs cocultured with CD4^+^ T cells significantly induced the release of IFN-γ via IL-12. BMDNs stimulated with eCIRP and IFN-γ underwent hyper-NETosis. Stimulating human peripheral blood neutrophils with eCIRP also induced APANs, and stimulating human neutrophils with eCIRP and IFN-γ caused hyper-NETosis. Thus, eCIRP released during sepsis induced APANs to aggravate ALI and worsen the survival of septic animals via CD4^+^ T cell activation, Th1 polarization, and IFN-γ–mediated hyper-NETosis.

## Graphical abstract

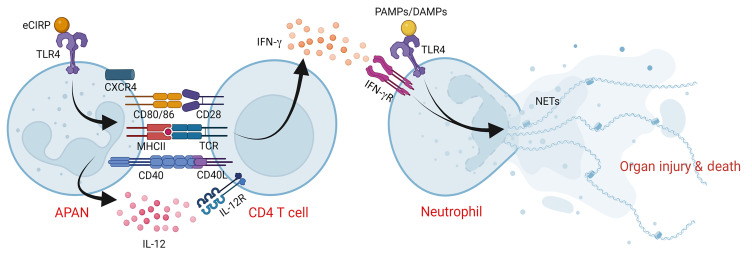

## Introduction

Sepsis is a life-threatening condition caused by an exaggerated immune response to infection (1). Neutrophils are the primary responders to infection, and neutropenic individuals are particularly vulnerable to sepsis (2), but uncontrolled activation of neutrophils also exacerbates inflammation and tissue injury, decisively contributing to the high lethality of sepsis (3). Although neutrophils had been previously thought to be terminally differentiated and short-lived, a growing body of evidence has revealed various neutrophil subtypes or states that are functionally diverse in critical immune phenotypes, such as their release levels of cytokines, myeloperoxidase, ROS, and neutrophil extracellular traps (NETs) in various pathological conditions (4–6).

Damage-associated molecular patterns (DAMPs) released by cells exposed to pathogens or hypoxia are key drivers of neutrophil heterogeneity, potentially worsening outcomes in sepsis (6, 7). Cold-inducible RNA-binding protein (CIRP) is an RNA chaperone that normally resides in the nuclear compartment (8). However, when cells are exposed to LPS or hypoxia, CIRP migrates to the cytoplasm and is released to the extracellular space via the lysosome-exocytosis pathway, exosomes, and inflammasome-mediated gasdermin-D pores to act as a DAMP (9–11). Extracellular CIRP (eCIRP) augments inflammation by binding to TLR4 on macrophages and neutrophils (9, 12). Using CIRP^–/–^ mice, we previously identified eCIRP as a critical mediator to aggravate organ injury and mortality in rodent models of sepsis. We have shown that CIRP^–/–^ mice exhibit significantly lower levels of serum organ injury markers and proinflammatory cytokines (13), reduced acute lung injury (13), and improved survival rates (14) compared with WT mice. Importantly, serum levels of eCIRP were later found to correlate with sepsis severity and mortality in humans as well (9, 15).

Emerging evidence shows that under certain conditions neutrophils can provoke antigen-specific T cell responses similar to those elicited by macrophages and dendritic cells (16, 17). We have shown that eCIRP released during sepsis increases neutrophil reverse transmigration as well as neutrophil surface expression of CXCR4 (18, 19) — a phenotypic marker of aged neutrophils that acts as a homing receptor to the bone marrow and secondary lymphoid organs. Therefore, we hypothesized that eCIRP aggravates sepsis by promoting a population consisting of antigen-presenting aged neutrophils (APANs). Here, we examined whether eCIRP induces the formation of APANs, the mechanism by which APANs aggravate sepsis, and the effect of these cells on sepsis severity.

## Results

### eCIRP promotes APAN generation.

**Figure 1:**
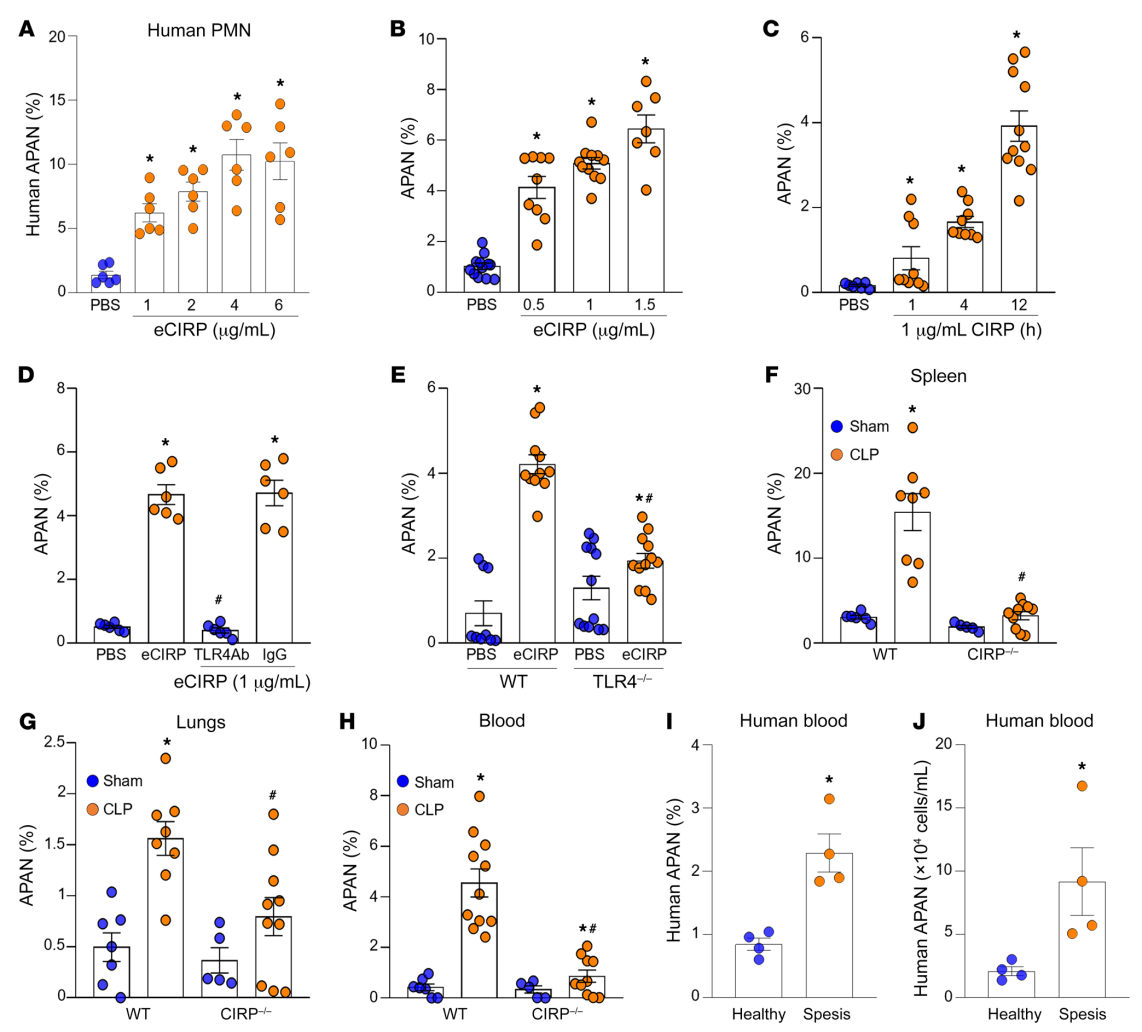
eCIRP generates APANs. (**A**) Human peripheral blood neutrophils (1 × 10^6^/mL) were treated with eCIRP (recombinant mouse CIRP) for 4 hours. The frequency of APANs (CD66b^+^CXCR4^+^CD62L^lo^CD40^+^CD86^+^) was assessed by flow cytometry. Data reflecting ≥3 experiments are expressed as mean ± SEM and compared by 1-way ANOVA and SNK test. *n =* 6/group. *P < 0.05 vs. PBS. (**B** and **C**) Mouse BMDNs (1 × 10^6^/mL) were treated with eCIRP (recombinant mouse CIRP) for various (**B**) doses and (**C**) time. The frequency of APANs (Ly6G^+^CXCR4^+^CD62L^lo^CD40^+^CD80^+^) was assessed by flow cytometry. Experiments were performed 3 times, and all data were analyzed. Data are expressed as mean ± SEM and compared by 1-way ANOVA and SNK test. *n =* 7–12/group. *P < 0.05 vs. PBS. (**D**) Mouse BMDNs (1 × 10^6^/mL) were treated with IgG/anti-TLR4 Ab (1 μg/mL of each) 30 minutes before stimulation with eCIRP (1 μg/mL) for 12 hours, followed by the detection of APANs by flow cytometry. Data are expressed as mean ± SEM and compared by 1-way ANOVA and SNK test. *n* = 6/group. *P < 0.05 vs. PBS, ^#^*P* <0.05 vs. eCIRP. (**E**) Mouse BMDNs (1 × 10^6^/mL) collected from WT and TLR4^–/–^ mice were treated with eCIRP (1 μg/mL) for 12 hours, followed by the detection of APANs by flow cytometry. Data reflecting ≥3 experiments are expressed as mean ± SEM and compared by 1-way ANOVA and SNK test. *n =* 6–12/group. *P < 0.001 vs. PBS/WT PBS; ^#^*P* < 0.05 vs. eCIRP/WT eCIRP. (**F**–**H**) Sepsis was induced in WT and CIRP^–/–^ mice by CLP. After 20 hours, the frequencies of APANs in Ly6G^+^ cells in the spleen, lungs, and blood were assessed by flow cytometry. Data reflecting ≥3 experiments are expressed as mean ± SEM and compared by 1-way ANOVA and SNK test. *n =* 5–11/group. *P < 0.05 vs. WT/CIRP^–/–^ sham; ^#^*P* < 0.05 vs. WT CLP. (**I** and **J**) Blood samples were collected from patients with sepsis and healthy individuals. Flow cytometry was used to determine the (**I**) frequency and (**J**) numbers of APANs (CD66b^+^CXCR4^+^CD62L^lo^CD40^+^CD86^+^). Data are expressed as mean ± SEM and compared by unpaired 2-tailed Student’s *t* test. *n =* 4/group. *P < 0.05 vs. healthy individuals.

To evaluate whether eCIRP can induce APANs, we first treated neutrophils isolated from healthy individuals’ whole-blood samples with eCIRP and assessed the surface expression of the antigen-presenting cell (APC) markers CD40 and CD86 and the aged neutrophil markers CXCR4 and CD62L in CD66b^+^ (pan neutrophil marker) cells. Human APANs were defined based on the surface phenotype CD66b^+^CXCR4^+^CD62L^lo^CD40^+^CD86^+^ (Figure 1A and Supplemental Figure 1A; supplemental material available online with this article; https://doi.org/10.1172/JCI164585DS1), of which more than 82% expressed HLA-DR (Supplemental Figure 1A). APANs had a higher expression of HLA-DR compared with other neutrophil subsets, namely non-aged antigen-presenting neutrophils (naAPNs) and non-antigen-presenting non-aged neutrophils (nAPANs) (Supplemental Figure 1B). eCIRP stimulation of human peripheral blood neutrophils significantly increased the frequency of APANs in a dose-dependent manner (Figure 1A). We next exposed murine bone marrow–derived neutrophils (BMDNs) to eCIRP and evaluated changes in the surface expression of the APC markers CD40 and CD86 and the aged neutrophil markers CXCR4^+^ and CD62L^lo^ in Ly6G^+^ cells. eCIRP significantly increased the frequency (percentage) of APANs in a dose- and time-dependent manner (Figure 1, B and C, and Supplemental Figure 1, C and D). APANs also expressed MHC-II at higher frequencies than other neutrophils (Supplemental Figure 1E). Wright/Giemsa-stained APANs had multilobed nuclei, rather than banded nuclei, indicating that these cells are activated mature neutrophils (Supplemental Figure 1F). To investigate whether eCIRP is a distinct inducer of APANs compared with other TLR4 ligands, we examined the effect of LPS, a commonly used TLR4 agonist, on APAN generation. LPS was able to induce APANs, but the magnitude of its induction was much lower than that with eCIRP stimulation. Treatment of BMDNs with LPS resulted in only a 2-fold increase in the APAN population (Supplemental Figure 1G). These findings support eCIRP as a potent TLR4 ligand driving APAN formation.

eCIRP-mediated induction of APANs was abrogated by TLR4-neutralizing Ab and in BMDNs from TLR4^–/–^ mice (Figure 1, D and E), indicating that eCIRP induces APAN expansion via TLR4 activation. In WT mice, APANs were significantly expanded in the spleen, lungs, and blood of septic mice compared with sham mice (Figure 1, F–H). However, in CIRP^–/–^ septic mice, we found that the frequency of APANs in the spleen, lungs, and blood was significantly decreased (Figure 1, F–H), further supporting eCIRP’s role in the induction of APANs in sepsis. These results indicate that eCIRP generates APANs in sepsis via TLR4.

Previously, we identified increased levels of eCIRP in the serum of patients with sepsis (9, 20). To establish the presence of APANs in humans, we examined human blood samples from patients with sepsis (Supplemental Table 1) and individuals in the healthy control group. Our findings indicated a significant increase in the percentage and number of APANs in the blood of patients with sepsis as compared with that in individuals in the control group (Figure 1, I and J). These data indicate that sepsis causes an increase in APANs in both mice and in humans.

### APANs constitute a phenotypically distinct neutrophil population.

**Figure 2:**
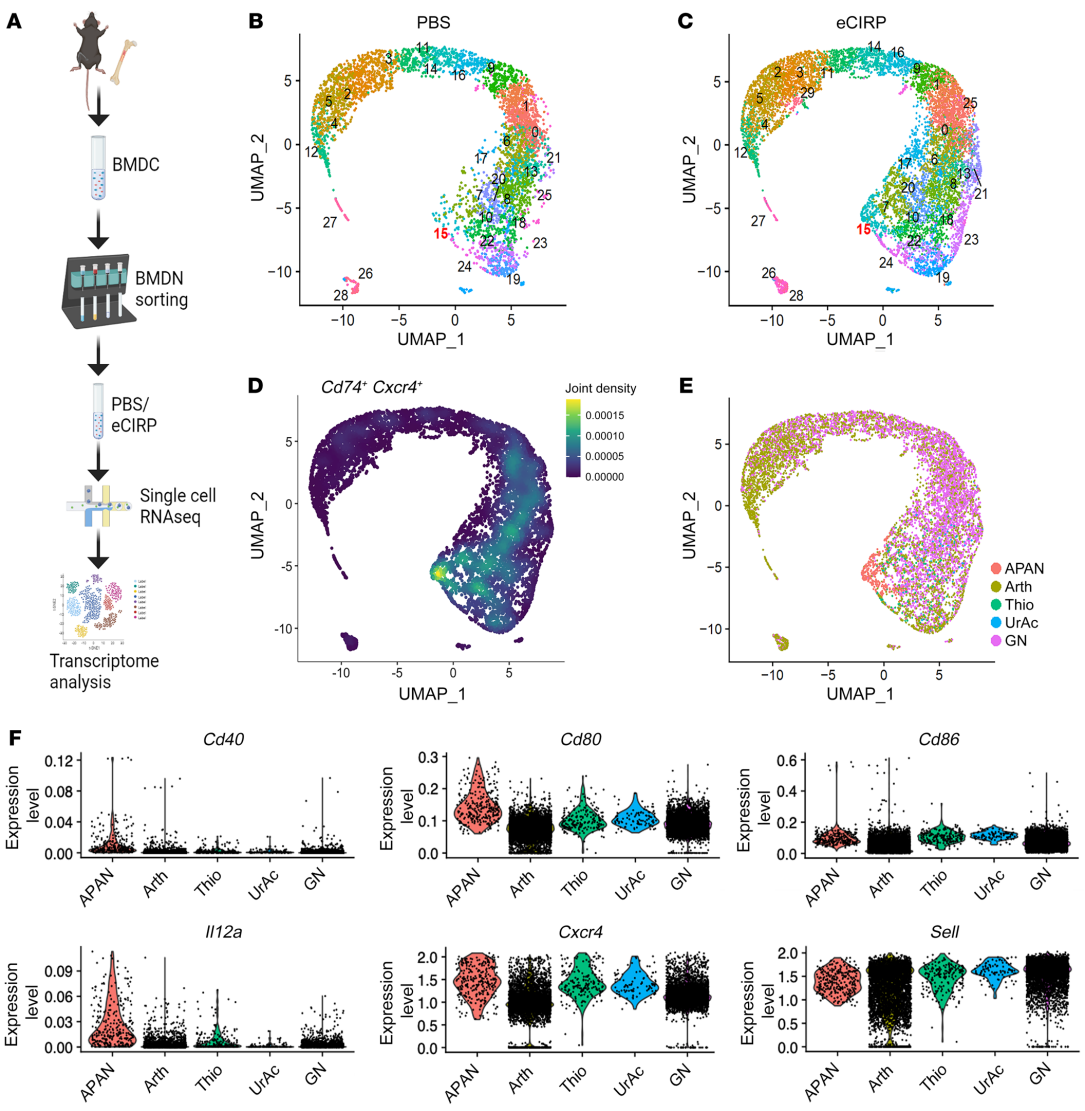
APANs constitute a phenotypically distinct neutrophil population. (**A**) After magnetic cell sorting purification, BMDNs (1 × 10^6^) were treated with eCIRP (1 μg/mL) for 2 hours and the cells were then processed through the 10× Genomics scRNA-Seq pipeline (i.e., barcoding and cDNA library preparation) for sequencing. (**B** and **C**) UMAP plot showing the results of postfiltering unsupervised random forest classification of (**B**) resting (PBS) and (**C**) eCIRP-stimulated BMDNs. (**D**) Cluster 15 had the highest coexpression of *Cd74* and *Cxcr4*. (**E**) UMAP plot reclustered using the ImmGen transcriptomes of mouse blood neutrophils (GN), neutrophils from arthritic mice (Arth), and peritoneal neutrophils stimulated in vivo with thioglycolate (Thio) or uric acid (UrAc). (**F**) Violin plots showing differential expression of key APAN marker genes across different neutrophil transcriptomes.

To further characterize eCIRP-induced APANs, we performed single-cell RNA-Seq (scRNA-Seq) on mouse BMDNs treated in vitro with PBS (as control) or eCIRP, processed via the 10× Genomics scRNA-Seq pipeline (Figure 2A). After quality control and filtering out nonneutrophils (Supplemental Figure 2, A–D), a distinct unsupervised cluster of APANs defined by coexpression of *Cd74* and *Cxcr4*, as well as induction by eCIRP stimulation, could be identified (Figure 2, B–D, and Supplemental Figure 2E). As shown in Figure 2D, *Cd74-* and *Cxcr4*-coexpressing cells were concentrated at the location corresponding to cluster 15 (shown in Figure 2, B and C). In addition, cluster 15 was clearly expanded after eCIRP stimulation (Figure 2, B and C and Supplemental Figure 2E). Thus, we identified cluster 15 as APANs. Next, we compared APANs with reference murine neutrophil transcriptomes available in the ImmGen database (Figure 2, E and F, and Supplemental Figure 2F). The reference mouse neutrophil transcriptomes consisted of unstimulated circulating neutrophils (GN), neutrophils from arthritic mice (Arth), and activated peritoneal neutrophils stimulated in vivo with thioglycolate (Thio) or uric acid (UrAc) (21). APANs expressed *Cd80* and *Il12a* at the highest levels among the neutrophil references (Figure 2F). *Cd86* levels were relatively higher in APANs, Thio, and UrAc and lower in Arth and GN (Figure 2F). The relatively high expression of *Cd86* not only in APANs but also in Thio and UrAc suggests that some of the costimulatory molecules of APCs are also upregulated by other forms of neutrophil activation. Nonetheless, all the costimulatory markers listed here were higher in APANs compared with GN, supporting their antigen-presenting phenotype.

### APANs produce increased levels of IL-12 in vitro and in vivo.

**Figure 3:**
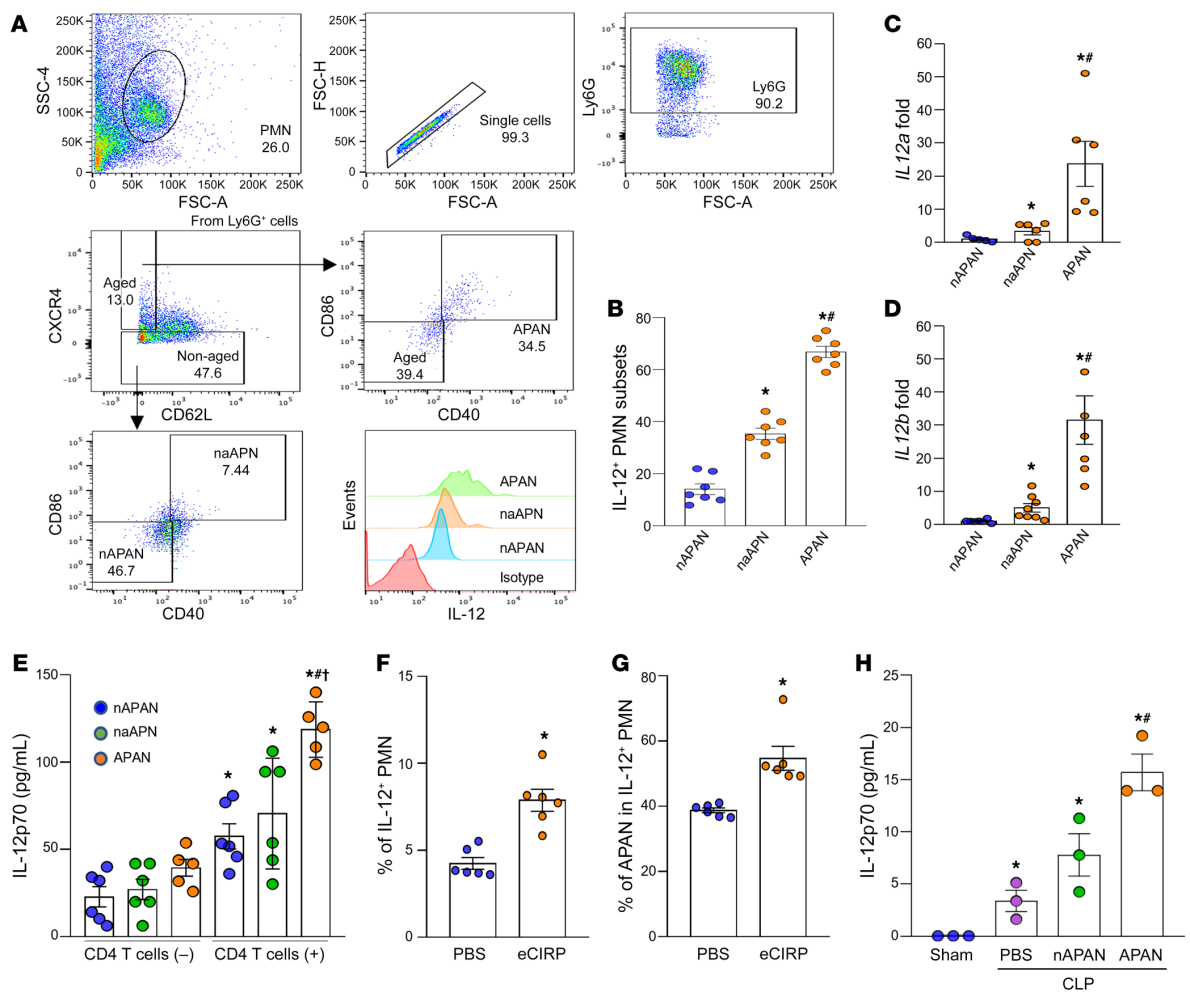
APANs produce high levels of IL-12. (**A** and **B**) BMDNs (1 × 10^6^) were treated with eCIRP (1 μg/mL) and brefeldin A (5 μg/ml). After 12 hours of stimulation with eCIRP, IL-12 expression in nAPANs (Ly6G^+^CXCR4^–^CD40^–^CD80^–^), naAPNs (Ly6G^+^CXCR4^–^CD40^+^CD80^+^), and APANs (Ly6G^+^CXCR4^+^CD62L^lo^CD40^+^CD80^+^) was determined by flow cytometry. Data reflecting 3 experiments are expressed as mean ± SEM. *n =* 7/group. *P < 0.05 vs. nAPAN, ^#^*P* < 0.05 vs. naAPN. (**C** and **D**) BMDNs (1 × 10^7^) were stimulated with eCIRP (1 μg/mL/10^6^ BMDNs) for 6 hours, and nAPANs, naAPNs, and APANs were sorted by flow cytometry. (**C**) *IL12a* and (**D**) *IL12b* mRNA was determined by real-time PCR. Data reflecting ≥3 experiments are expressed as mean ± SEM. *n =* 6/group. *P < 0.05 vs. nAPAN, *P* < 0.05 vs. naAPN. (**E**) BMDNs (1 × 10^7^) were stimulated with eCIRP (1 μg/mL/10^6^ BMDNs) for 6 hours, and nAPANs, naAPNs, and APANs sorted by flow cytometry were cocultured with CD4^+^ T cells (1:1 ratio) for 12 hours. IL-12p70 levels in the culture supernatants were assessed by ELISA. Data from ≥3 experiments are expressed as mean ± SEM. *n =* 5–6/group. *P < 0.05 vs. nAPAN without T cells, ^#^*P* < 0.05 vs. nAPAN with T cells, ^†^*P* < 0.05 vs. naAPN with T cells. (**F** and **G**) Mice were injected with eCIRP (5 mg/kg, i.v.), and 4 hours later, the frequencies of IL-12^+^ cells, PMNs, and APANs were assessed by flow cytometry. (**F**) Frequency of IL-12^+^ neutrophils and (**G**) of APANs in IL-12^+^ cells are shown. Data reflecting ≥3 experiments are expressed as mean ± SEM. *n =* 6/group. *P < 0.05 vs. PBS. (**H**) nAPANs or APANs (1 × 10^6^) FACS-sorted from mouse BMDNs stimulated with eCIRP (1 μg/mL for 6 hours) were i.v. injected into mice immediately after CLP. 20 hours after CLP, the serum levels of IL-12p70 were assessed by ELISA. Data are expressed as mean ± SEM. *n =* 3/group. *P < 0.05 vs. sham, ^#^*P* < 0.05 vs. PBS+CLP, ^†^*P* < 0.05 vs. nAPAN+CLP. Data were compared by 1-way ANOVA and SNK test.

IL-12 is a cytokine produced by APCs during antigen presentation that promotes type-1 Th1 cells. After 12 hours of stimulation with eCIRP, APANs contained a significantly higher amount of intracytoplasmic IL-12 compared with nAPANs and naAPNs (Figure 3, A and B). We also sorted APANs, nAPANs, and naAPNs from the eCIRP-stimulated BMDNs and assessed the mRNA expression of IL-12a and IL-12b by qPCR. Like the IL-12 protein expression data, both IL-12a and IL-12b mRNA levels were significantly increased in APANs compared with nAPANs and naAPNs (Figure 3, C and D). Next, we cocultured with CD4^+^ T cells isolated from OT-II transgenic mice with APANs, nAPANs, and naAPNs preloaded with an MHC-II–restricted OVA peptide epitope and found that APANs produced significantly higher levels of IL-12 compared with nAPANs and naAPNs (Figure 3E). Naive mice injected with eCIRP generated significantly more IL-12–producing neutrophils, of which nearly 60% were APANs (Figure 3, F and G). Similarly, septic mice adoptively transferred APANs had significantly higher serum levels of IL-12 compared with septic mice injected PBS or nAPANs (Figure 3H). These data demonstrate that APANs release IL-12 during cognate antigen presentation to CD4^+^ T cells in sepsis.

### APANs promote CD4^+^ T cell proliferation, Th1 polarization, and IFN-γ release.

**Figure 4:**
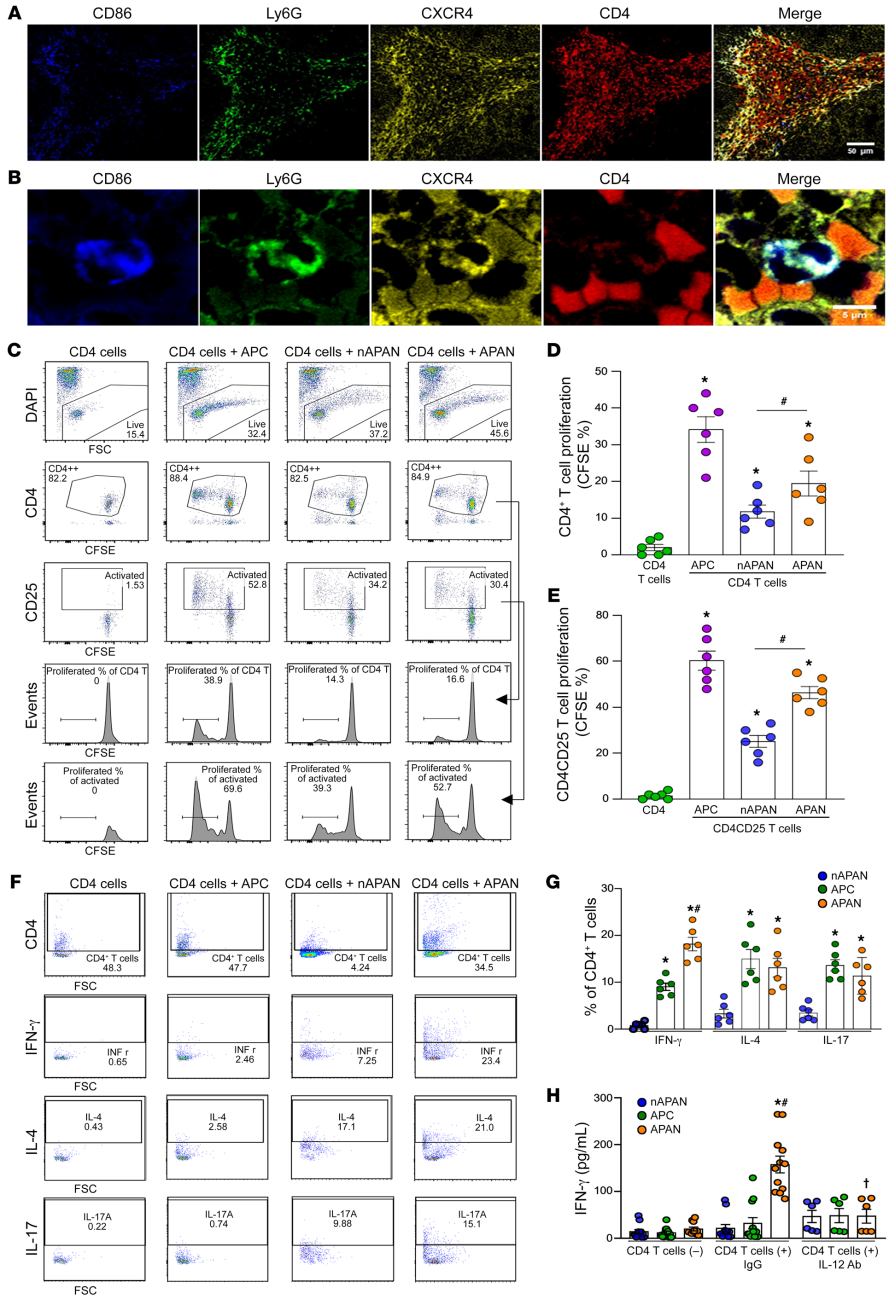
APANs promote CD4^+^ T cell proliferation and IFN-γ release. (**A** and **B**) Spleen tissue sections from WT septic mice (20-hour CLP) were stained with anti-Ly6G, CXCR4, CD86, and CD4 Abs. Representative images of immunohistochemical stains of spleen are shown. Original magnification, ×20 (**A**); ×200 (**B**). Scale bar: 50 μm (**A**); 5 μm (**B**). (**C**–**E**) WT mouse BMDNs (1 × 10^7^) were stimulated with eCIRP (1 μg/mL/10^6^ cells) for 6 hours. FACS-purified APANs, nAPANs, and naive splenic F4/80^+^ macrophage as antigen-presenting cell (APC) controls (1 × 10^5^) were cocultured with splenic CD4^+^ T cells (1 × 10^5^) isolated from naive OT-II transgenic mice. After 72 hours, the cells were stained with anti-CD4, and CD25 Ab, and CSFE dye. The proliferation of (**C** and **D**) total CD4^+^ T cells or (**C** and **E**) CD4^+^CD25^+^ T cells was assessed. (**C**) Representative flow cytometry gating of the CD4^+^ T cell proliferation. (**D** and **E**) Frequency of total CD4^+^ T cell or CD4^+^CD25^+^ T cell proliferation. Data reflecting ≥3 independent experiments are expressed as mean ± SEM and compared by 1-way ANOVA and SNK test. *n =* 6/group. *P < 0.05 vs. CD4^+^ T cells only, ^#^*P* < 0.05 vs. nAPAN^+^CD4^+^ T cells. (**F** and **G**) APANs, nAPANs, or APCs (1 × 10^5^) were cocultured with OT-II transgenic mouse splenic CD4^+^ T cells (1 × 10^5^), and, 24 hours later, IFN-γ, IL-4, and IL-17 culture supernatant levels were determined by ELISA. Data reflecting ≥3 independent experiments are expressed as mean ± SEM and compared by ANOVA and SNK test. *n =* 6. *P < 0.05 vs. nAPAN, ^#^*P* < 0.05 vs. APC. (**H**) APANs, nAPANs, or APCs (1 × 10^5^) were cocultured with OT-II mouse splenic CD4^+^ T cells (1 × 10^5^) in the presence of IgG or IL-12– neutralizing Ab. At 24 hours later, IFN-γ culture supernatant levels were determined by ELISA. Data reflecting ≥3 independent experiments are expressed as mean ± SEM and compared by ANOVA and SNK test. *n =* 6–12/group. *P < 0.05 vs. nAPAN, ^#^*P* < 0.05 vs. APC, ^†^*P* < 0.05 vs. APAN+IgG.

Neutrophils have been shown to interact with lymphocytes both at the site of inflammation and in secondary lymphoid organs (22, 23). To elucidate how APANs interact with CD4^+^ T cells, we first evaluated their colocalization by immunostaining. APANs and CD4^+^ T cells were present in direct contact in the marginal zones of the spleen of septic mice (Figure 4, A and B). We next stimulated BMDNs with eCIRP and sorted APANs and nAPANs. We also isolated mouse splenic macrophages to serve as professional APC^+^ controls. We then cocultured each of these cells with CD4^+^ T cells isolated from OT-II transgenic mice preloaded with an MHC-II–restricted OVA peptide epitope. While all cells promoted CD4^+^ T cell proliferation, APANs induced significantly more CD4^+^ T cell proliferation than nAPANs, although less than professional APCs (Figure 4, C and D). The same results were observed when we restricted the analysis to activated CD4^+^ T cells (CD4^+^CD25^+^) (Figure 4, C and E). Compared with nAPANs and APCs, APANs were particularly effective in inducing IFN-γ–producing Th1 cells (Figure 4, F and G). APANs were also similar to APCs and significantly better than nAPANs at inducing IL-4– and IL-17–producing Th2 and Th17 cells, respectively (Figure 4, F and G). In the presence of IgG control, CD4^+^ T cells cocultured with APANs released significantly more IFN-γ than when cocultured with nAPANs or APCs, and IL-12–neutralizing Ab completely abrogated the release of APAN-induced IFN-γ (Figure 4H). These results demonstrate that APANs promote CD4^+^ T cell proliferation as well as Th1 differentiation and IFN-γ release via IL-12.

We found that APANs produced higher levels of IL-12 than nAPANs and naAPNs (Figure 3, A–D), findings that support those of previous studies identifying IL-12 as a potential driver of Th1 differentiation (24). We noticed that some of the CD4^+^ T cells exposed to APANs had positive intracytoplasmic staining for IL-4 and IL-17 (Figure 4, F and G). To further explore this, we examined the expression of IL-4 (a cytokine involved in Th2 differentiation) and IL-21 (a cytokine for Th17 differentiation) in the APAN population using our scRNA-Seq data. Our analysis, as shown in Supplemental Figure 2, G and H, revealed no upregulation of these cytokines (no reads were detected to these genes) in the APAN cluster.

### CD4^+^ T cell and APAN interaction leads to increased NET formation via IFN-γ.

**Figure 5:**
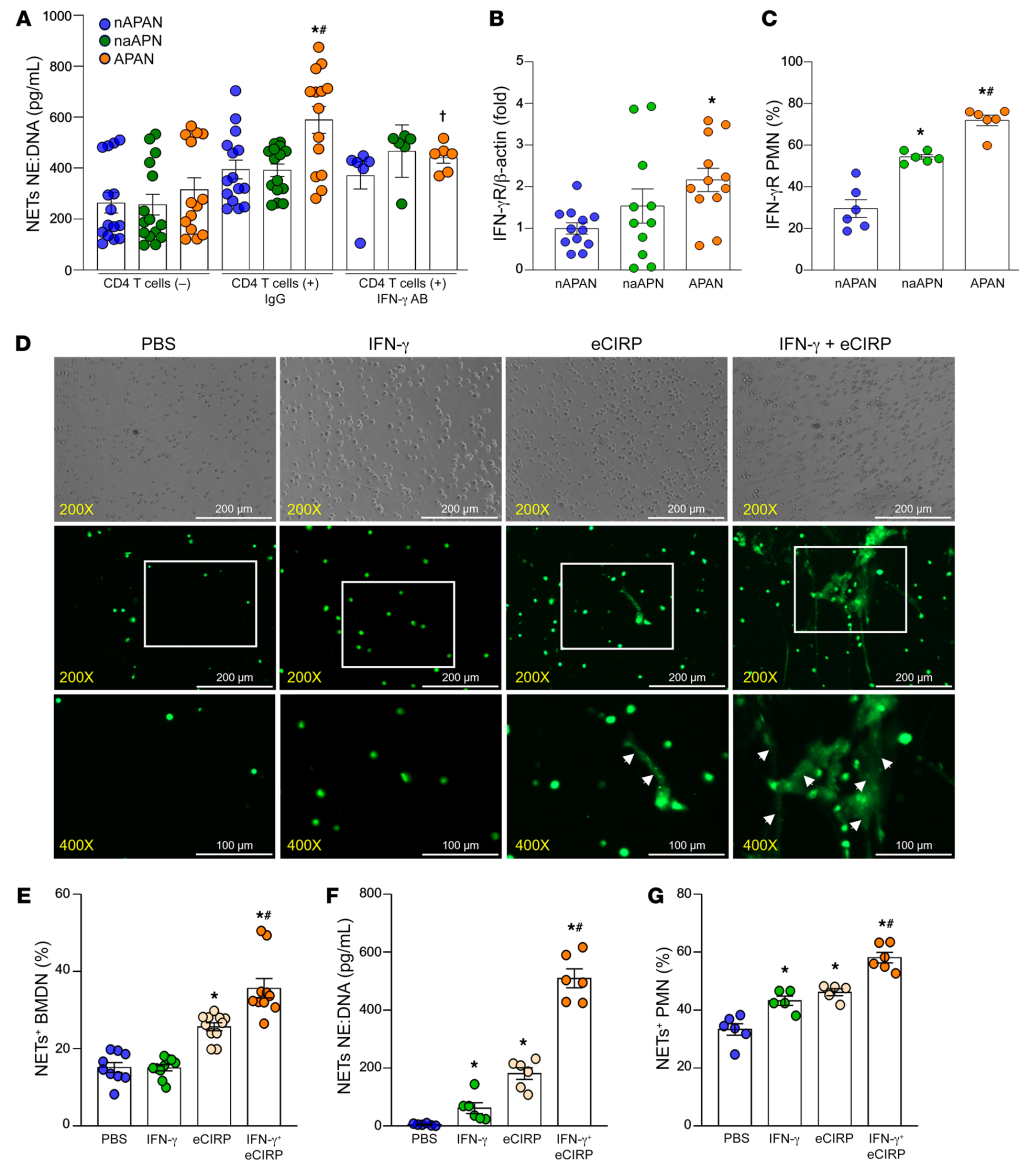
APAN-activated CD4^+^ T cells promote NET formation via IFN-γ. (**A**) BMDNs (1 × 10^7^) were stimulated with eCIRP (1 μg/mL/10^6^ cells) for 6 hours. FACS-isolated APANs, nAPANs, and naAPNs (1 × 10^6^ cells/mL) were cultured with or without mouse splenic CD4^+^ T cells (1 × 10^6^ cells/mL) in 48-well plates (200 μL, final volume) in the presence of IgG or IFN-γ–neutralizing Ab. After 4 hours, culture supernatant NETs were assessed by ELISA. Data reflecting ≥3 independent experiments are expressed as mean ± SEM and compared by ANOVA and SNK test. *n =* 6–15/group. *P < 0.05 vs. nAPAN only, ^#^*P* < 0.05 vs. nAPAN+CD4^+^ T cells, ^†^*P* < 0.05 vs. APAN+CD4^+^ T cells+IgG. (**B**) The mRNA expression of IFN-γR in APANs, nAPANs, and naAPNs was assessed by real-time qPCR. (**C**) APANs, nAPANs, naAPNs were stained with anti–IFN-γR Ab and then assessed IFN-γR expression by flow cytometry. Data reflecting ≥3 independent experiments are expressed as mean ± SEM and compared by ANOVA and SNK test. *n =* 6–12/group. *P < 0.05 vs. nAPAN, ^#^*P* < 0.05 vs. naAPN. (**D**–**F**) Mouse BMDNs (1 × 10^6^ cells/mL) were stimulated with IFN-γ (10 ng/mL) with or without eCIRP (1 μg/mL) and, 4 hours later, assessed for NET formation using 3 methods: (**D**) fluorescent microscopy by staining the cells with Sytox Green, where the white arrows indicate the NET-like structures (original magnification, ×200 [rows 1 and 2]; ×400 [row 3]; scale bar: 200 μm [rows 1 and 2]; 100 μm [row 3]) (*n =* 6–9/group); (**E**) flow cytometry by staining unpermeabilized cell for myeloperoxidase (MPO) and citH3 Ab (*n =* 9–11/group); and (**F**) ELISA using neutrophil elastase (NE) and anti-dsDNA Ab (*n =* 6/group). (**G**) Isolated human neutrophils (1 × 10^6^ cells/mL) were stimulated with IFN-γ (10 ng/mL) with and without eCIRP (1 μg/mL), and, 4 hours later, NET formation was assessed by flow cytometry by staining unpermeabilized neutrophils with anti-human MPO and citH3 Ab. Data are expressed as mean ± SEM and compared by 1-way ANOVA and SNK test. *n =* 6/group. *P < 0.05 vs. PBS, ^#^*P* < 0.05 vs. eCIRP.

To evaluate the effects of the CD4^+^ T cell and APAN interaction on neutrophils, we cocultured OVA peptide-loaded nAPANs, naAPNs, and APANs with CD4^+^ T cells from OT-II transgenic mice and assessed NET formation. In the presence of IgG control and CD4^+^ T cells, APANs increased NET formation by 87% compared with APAN cultures without CD4^+^ T cells (Figure 5A), indicating that CD4^+^ T cells play a critical role in APAN-induced NET formation. Interestingly, IL-12–neutralizing Ab significantly diminished APAN-induced NET formation compared with IgG control (Figure 5A). Because APAN-released IL-12 induced IFN-γ production by CD4^+^ T cells (Figure 4H), we focused on IFN-γ’s effect on APANs. Indeed, APANs expressed significantly higher mRNA and surface protein levels of IFN-γ receptor (IFN-γR) compared with nAPANs and naAPNs (Figure 5, B and C). To further elucidate IFN-γ’s role in NET formation, we stimulated BMDNs with recombinant mouse IFN-γ (rmIFN-γ), eCIRP, or rmIFN-γ plus eCIRP. Surprisingly, we found that BMDNs stimulated with IFN-γ plus eCIRP produced more NET-like structures (Figure 5D, as indicated by the arrowheads), compared with those treated with eCIRP alone. To quantitate NETs, we also assessed NETs by flow cytometry and ELISA, which showed a significant increase in NET formation in BMDNs stimulated with IFN-γ plus eCIRP compared with only IFN-γ or only eCIRP (Figure 5, E and F). We further confirmed this finding in human peripheral blood neutrophils. Human peripheral blood neutrophils stimulated with eCIRP and recombinant human IFN-γ (rhIFN-γ) in combination showed a significant increase in the NET formation compared with only IFN-γ or only eCIRP (Figure 5G). These data show that APAN-stimulated CD4^+^ T cells increase NET formation via IFN-γ in the presence of eCIRP.

### APANs worsen inflammation, lung injury, and survival in sepsis.

**Figure 6:**
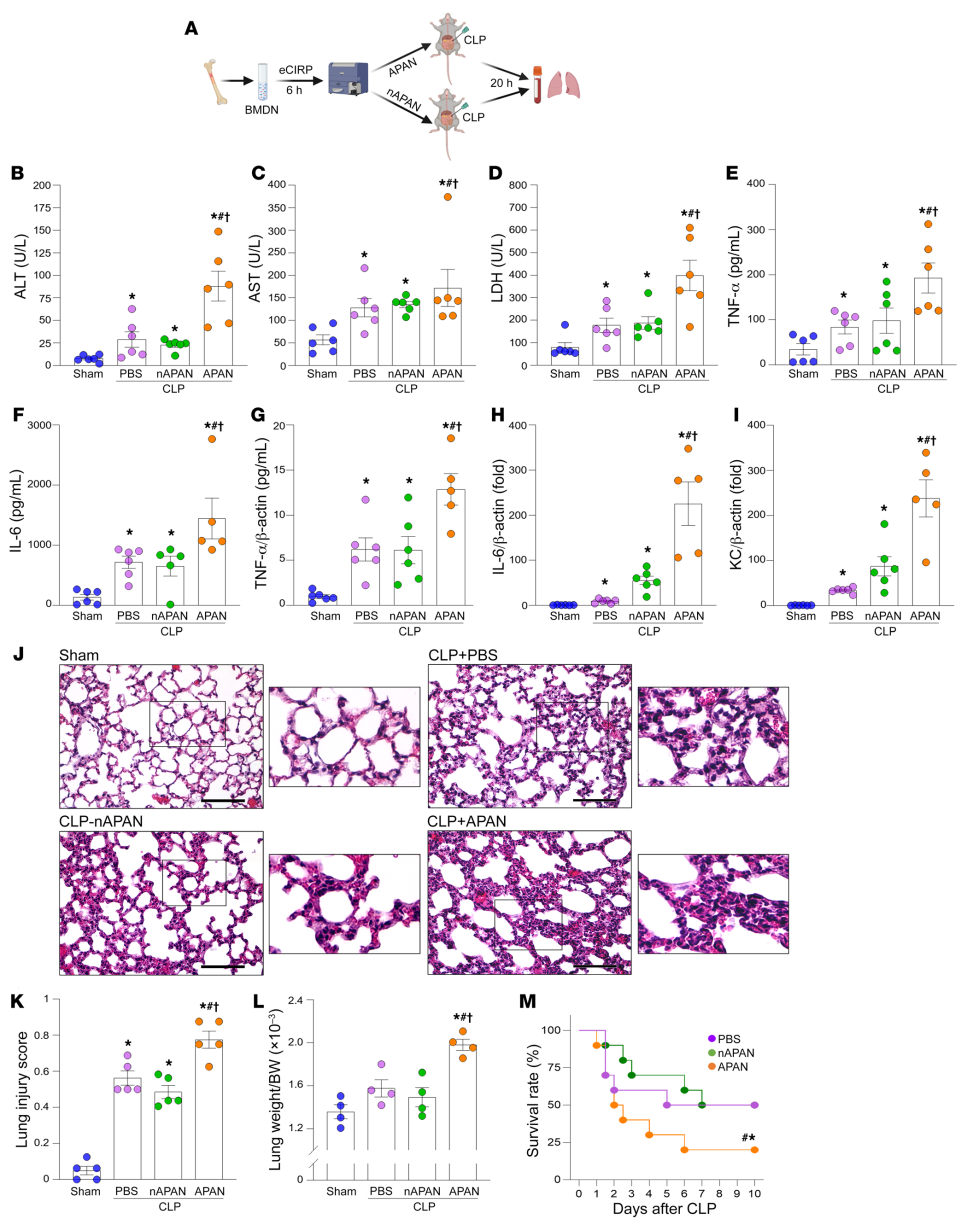
APANs exaggerate sepsis by fueling inflammation, lung injury, and worsening survival. (**A**) BMDNs isolated from WT mice were stimulated with eCIRP for 6 hours. FACS-isolated APANs and nAPANs (1 × 10^6^) were then adoptively transferred into mice via retro-orbital injection at the time of CLP. (**B**–**I**) Twenty hours later, the serum levels of (**B**) ALT, (**C**) AST, and (**D**) LDH were determined using specific colorimetric enzymatic assays; serum (**E**) TNF-α and (**F**) IL-6 levels were assessed by ELISA; and lung mRNA levels of (**G**) TNF-α, (**H**) IL-6, and (**I**) KC were assessed by real-time PCR. Data are expressed as mean ± SEM (*n =* 5–6 mice/group) and compared by ANOVA and SNK method. *P < 0.05 vs. sham, ^#^*P* < 0.05 vs. CLP+PBS-treated mice, ^†^*P* < 0.05 vs. CLP+nAPAN-injected mice. (**J**) Representative images of H&E-stained lung tissue. Original magnification, ×400. Scale bar: 100 μm. Enlarged images of the boxed areas are shown to the right. (**K**) Lung injury score. Average of 5 fields/slide/mouse. Data are expressed as mean ± SEM (*n =* 5 mice/group) and compared by ANOVA and SNK method. *P < 0.05 vs. sham, ^#^*P* < 0.05 vs. CLP+PBS-treated mice, ^†^*P* < 0.05 vs. CLP+nAPAN-injected mice. (**L**) Wet lung weight–to–body weight (BW) ratio 20 hours after CLP. Data are expressed as mean ± SEM (*n =* 4 mice/group) and compared by ANOVA and SNK method. *P < 0.05 vs. sham, ^#^*P* < 0.05 vs. CLP+PBS-treated mice, ^†^*P* < 0.05 vs. CLP+nAPAN-injected mice. (**M**) Kaplan-Meier 10-day survival curve generated from PBS-, APAN-, and nAPAN-treated CLP mice. *n =* 20 mice/group, *P < 0.05 vs. CLP+PBS, ^#^*P* < 0.05 vs. CLP+nAPAN-injected mice determined by the log-rank test.

To determine how APANs affect inflammation and tissue injury in sepsis, we adoptively transferred APANs or nAPANs to mice at the time of cecal ligation and puncture (CLP) induction and collected blood and lungs 20 hours later (Figure 6A). Compared with PBS- or nAPAN-injected septic mice, APAN-injected septic mice had significantly higher serum levels of hepatic and cell injury markers (ALT, AST, and LDH) and proinflammatory cytokines (TNF-α, IL-6), as well as increased lung TNF-α, IL-6, and KC mRNA expression (Figure 6, B–I). The lungs of APAN-injected septic mice exhibited increased alveolar congestion, proteinaceous debris, interstitial and alveolar neutrophil infiltration, alveolar hemorrhage, and damage to epithelial architecture (Figure 6, J and K). To evaluate the extent of pulmonary edema following sepsis, we measured the wet weight of the lungs and body weight (BW). Our findings demonstrated that while the wet lung weight–to-BW ratio was slightly higher in sepsis mice than in sham mice, there was a significant increase in this ratio among sepsis mice receiving APANs compared with those receiving PBS or nAPANs (Figure 6L). This indicates that APANs can worsen pulmonary edema during sepsis. Importantly, compared with PBS- and nAPAN-injected septic mice, APAN-injected septic mice had significantly decreased 10-day survival after CLP (Figure 6M). To determine the isolated, direct effects of APAN, in a pilot study, we subjected neutropenic mice (PMN^DTR^ mice) adoptively transferred with either APANs or nAPANs to an attenuated model of sepsis (otherwise, the neutropenic mice died). Following the induction of neutropenia, we observed a significant reduction in neutrophil and white blood cell counts in the PMN^DTR^ mice, as compared with WT mice (Supplemental Table 2). Compared with PBS- or nAPAN-injected septic neutropenic mice, we found that APAN-injected septic neutropenic mice had significantly higher serum levels of ALT, AST, LDH, TNF-α, and IL-6, as well as increased lung TNF-α, IL-6, and MIP-2 mRNA expression (Supplemental Figure 3). Thus, APANs aggravate sepsis by worsening inflammation, leading to more severe lung injury and culminating in decreased survival.

## Discussion

**Figure 7:**
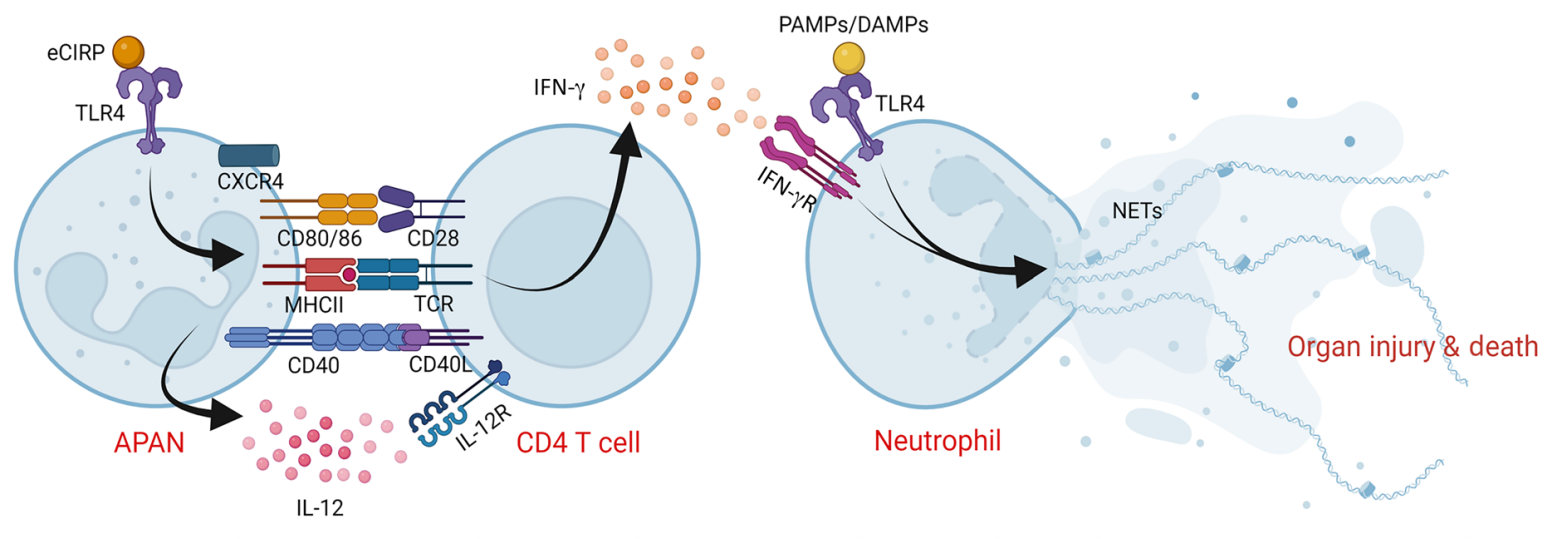
APANs exacerbate sepsis by inducing IFN-γ production in CD4^+^ T cells. eCIRP induces neutrophils to generate APANs (CXCR4^+^CD62L^–^CD40^+^CD86^+^MHCII^+^), which release high levels of IL-12. APANs present antigen to CD4^+^ T cells, inducing Th1 polarization and release of IFN-γ, which in turn primes neutrophils to undergo hyper-NETosis induced by eCIRP, ultimately, increasing inflammation and aggravating acute lung injury, thus worsening the mortality in sepsis.

In the present study, we have identified a distinct neutrophil subpopulation characterized by the presence of both antigen-presenting and aged surface phenotypic markers, which we named APANs. We have shown that eCIRP, a major proinflammatory mediator in sepsis, is a key inducer of APANs. We have also demonstrated that APANs engage in cognate antigen presentation with CD4^+^ T cells, resulting in the production of high levels of IL-12. The APAN-derived IL-12 then induces Th1 polarization and IFN-γ production, which, in turn, results in NET formation via the IFN-γ/IFN-γR pathway in APANs (and possibly by other bystander neutrophils as well as remotely located neutrophils, considering that APANs caused increased blood levels of IFN-γ in septic mice). As a result, these processes lead to augmented inflammation, aggravated acute lung injury, and increased mortality in sepsis (Figure 7).

Excessive neutrophil activation and function are critical causes of tissue damage and organ injury, which contributes to sepsis morbidity and mortality (6, 7). However, neutrophil-depleting strategies should not be employed for treating patients with sepsis because these approaches can markedly reduce the production of antimicrobial agents. Consequently, neutropenic patients may lack the necessary defenses to effectively combat infectious microorganisms (25). As such, the identification of deleterious neutrophil populations may allow the prevention of neutrophil-induced damage without causing immunosuppression. A number of neutrophil phenotypes have been described by recent studies, including ICAM-1^+^ neutrophils (26), CD11b^hi^ low-density neutrophils (19), and reverse-transmigrated neutrophils (18). Aged neutrophils are one of the most-studied neutrophil phenotypes; they are deleterious in sepsis and characterized by a CXCR4^hi^CD62L^lo^ immunophenotype (27). Aged neutrophils exert proinflammatory effects as they produce excess amounts of ROS and NETs (27).

Among many of the neutrophil functions, cognate antigen presentation is perhaps the least studied. Much like professional APCs, neutrophils can also induce antigen-specific T cell responses (16, 17). Neutrophils can be induced to express surface proteins critical for antigen presentation, i.e., MHC-II and costimulatory molecules (CD40, CD80, CD86) (16). Furthermore, neutrophils exposed to GM-CSF, IFN-γ, IL-3, and TNF-α acquire the ability to present antigens to CD4^+^ T cells (16, 28–30). Although the antigen-presenting and aged phenotypes have been identified separately (16, 27), neutrophils expressing both antigen-presenting and aged markers in combination had not yet been studied to our knowledge. While exploring the effects of eCIRP on neutrophil heterogeneity, we identified a small population of APANs characterized by the combined expression of antigen-presenting and aged immunophenotypic markers (Ly6G^+^CXCR4^+^CD62L^lo^CD40^+^CD80^+^MHCII^+^). APANs and CD4^+^ T cells colocalized in the spleen. Theoretically, this interaction would be even greater in sepsis, as neutrophils infiltrate into lymphoid tissues due to systemic inflammation, whereas few neutrophils can normally be observed in secondary lymphoid tissues in healthy animals. Further gene expression profile analysis showed that APANs express IL-12 at higher levels than either aged neutrophils or antigen-presenting neutrophils. IL-12 is a heterodimeric pleiotropic cytokine typically produced by professional APCs (i.e., macrophages and dendritic cells) in response to certain pathogens to promote Th1 polarization and IFN-γ production (31).

To investigate the antigen-presenting ability of APANs, we loaded them with OVA and evaluated their capacity to activate and differentiate CD4^+^ T cells from OT-II transgenic mice. We found that the antigen presentation of APANs markedly increased CD4^+^ T cell activation, proliferation, and Th1 differentiation, similar to splenic APCs, which served as a positive control. Our primary focus was to determine APAN’s ability to activate CD4^+^ T cells rather than comparing them with other neutrophil subtypes, like naAPNs. For CD4^+^ T cell activation, we chose splenic APCs, which are conventional APCs, and our results revealed that APANs could activate CD4^+^ T cells like the splenic APCs. While we did not investigate the antigen-presenting ability and CD4^+^ T cell activation of naAPNs, it is possible that, like nAPANs, the relatively lower levels of IL-12 expressed by naAPNs could lead to reduced effectiveness in activating and differentiating CD4^+^ T cells compared with APANs, as IL-12 is known to promote Th1 differentiation (24). While APANs derived from both peripheral blood neutrophils and BMDNs express identical immunophenotypic markers, we conducted antigen presentation and other functional studies using APANs generated from BMDNs ex vivo stimulated with eCIRP due to the higher recovery cell numbers. However, it’s worth noting that other factors, such as the neutrophil stage of maturation, the nature of the pathogens/cells/particles phagocytosed, and the microenvironment of the site where phagocytosis occurs, may influence the antigen presentation characteristics of individual APANs. Therefore, future studies could investigate the antigen-presenting ability of blood versus bone marrow–derived APANs to determine any functional differences.

In the present study, we found that eCIRP, a key proinflammatory mediator in sepsis, promotes APAN formation. Interestingly, our previous study showed that eCIRP also increases neutrophil expression of ICAM-1 (26), an adhesion molecule typically expressed by aged neutrophils (19, 27, 32). Neutrophil ICAM-1 expression correlates with enhanced phagocytosis, while ICAM-1 deficiency impairs phagocytic function in neutrophils (32). Because phagocytosis is an essential process leading to antigen presentation, this characteristic of aged neutrophils should enhance and facilitate antigen processing and presentation by APANs. We also found that eCIRP induces APANs in a TLR4-dependent manner. This is an interesting finding, considering that the microbiota has been shown to drive neutrophil aging via TLR4 (27). Interestingly, depleting the microbiota reduces the number of circulating aged neutrophils and dramatically attenuates sepsis severity (27). Moreover, the TLR4 pathway plays a critical role in sepsis, especially when initiated by Gram-negative bacteria, with LPS directly activating TLR4 on various immune and nonimmune cells (33). Once sepsis-induced cellular damage occurs, eCIRP, other DAMPs, and endotoxin can activate TLR4 to further exacerbate inflammation (34). Therefore, they may also play a role in APAN formation.

We not only identified and characterized APANs, but also assessed the effect of their adoptive transfer in sepsis. APANs aggravated proinflammatory markers and lung injury and decreased the survival of septic WT mice. We also utilized neutropenic mice, in which the influence of host neutrophils is minimized, to further study the effects of APANs in sepsis. Similar to that observed in WT mice, the adoptive transfer of APANs to neutropenic mice resulted in elevated levels of proinflammatory parameters and organ injury markers compared with neutropenic mice that received non-APANs. Because neutropenic mice are very susceptible to infection (35), we utilized a milder model of sepsis in these animals. In addition, neutrophils are critical players of innate immunity, and their deficiency is detrimental to sepsis; thus, mice transferred with nAPANs served as a control, and neutrophils were transferred in all groups of animals used in this experimental setting. A technical point could be raised regarding the potential effect on cell viability resulting from the extended process involved in isolating mouse neutrophils and flow sorting after the 6-hour incubation with eCIRP. However, we addressed this concern by using flow cytometry dot blots to analyze the gating strategy of the APANs generated from eCIRP-stimulated BMDNs. Our results revealed that after 4 hours of eCIRP stimulation, the number of dead cells or amount of debris was not significantly different from that at the 0-hour sample. Although the number of dead cells did increase after 12 hours of eCIRP stimulation, for the ex vivo generation of the APANs, we treated the BMDNs with eCIRP for 6 hours prior to sorting and excluded debris and dead cells in the FSC/SSC gating while sorting the APANs. After sorting the APANs, we immediately injected them into mice, where they were able to survive in a much more physiologic environment compared with ex vivo conditions. Because nonviable cells were excluded during sorting, we assume that the sorted cells retained their viability, as demonstrated by their deleterious role in sepsis. Furthermore, the sorted cells expressed an aged phenotype, which may contribute to a prolonged life span and activity in vivo.

During sepsis, NETs released from activated neutrophils cause inflammation and tissue damage (7). NETs contain antimicrobial enzymes, such as neutrophil elastase and myeloperoxidase, as well as DAMPs, including histone H3, cell-free DNA, HMGB1, and eCIRP (7). Some of these DAMPs, such as HMGB1 and eCIRP, can, in turn, induce NETosis to form a vicious cycle; thus, NET release needs to be tightly regulated. Here, we showed that NET formation induced by a DAMP, i.e., eCIRP, is significantly enhanced by IFN-γ released from CD4^+^ T cells. The fragile structure of NETs makes it challenging to perform extensive washing without loss of NETs. This may explain the high baseline signal in our flow cytometry–based assessments of NETs, resulting from incomplete removal of nonspecifically bound Abs. To address this limitation and provide additional support for the flow cytometry data, we also used ELISA and fluorescence microscopy. Taken together, these multiple detection methods provide a more rigorous confirmation of NET formation. NETs have been shown to induce T cell activation as well as apoptosis (36, 37), which may contribute to lymphopenia in the circulation and lymphoid tissues seen in sepsis (38). Thus, assessing the role of APANs on T cell apoptosis and lymphopenia would be of interest in future studies.

APANs are a distinct neutrophil population induced by eCIRP. Because there are no prior reports on this population to our knowledge, we first investigated its potential clinical relevance by assessing the presence of APANs in human blood. Our results showed a significant increase in blood frequency of APANs in patients with sepsis, suggesting the possibility of similar findings in other acute inflammatory conditions. Recent studies have shown increases in the blood levels of eCIRP (39) and in the lung content of NETs in patients with COVID-19 (40); this could be attributed to the presence of APANs, as these cells are potential drivers of NETosis through T cell activation. Interestingly, one of our patients with sepsis, who has increased APAN levels, was discovered to be COVID-19 positive, indicating the possibility that these cells can also be present due to COVID-19 infection. Future studies investigating the presence of APANs in patients with COVID-19 could shed light on the disease pathophysiology. In conclusion, we have identified a neutrophil population exhibiting both antigen-presenting and aged phenotypes, named APANs. APANs act by promoting cognate antigen presentation, CD4^+^ T cell activation, Th1 polarization, and IFN-γ–mediated hyperNETosis. APANs aggravate sepsis, worsen acute lung injury, and diminish the survival of septic animals, and their number in the circulation may serve as a biomarker of sepsis severity. Because APANs are induced by eCIRP, pharmacological strategies to inhibit eCIRP may be developed to target APANs and ameliorate sepsis.

## Methods

### Participants.

Blood samples were collected from deidentified patients with sepsis admitted to the surgical intensive care unit at North Shore University Hospital, which is part of Northwell Health (New Hyde Park, New York, USA), as well as from healthy individuals in the control group.

### Isolation of human neutrophils and stimulation with eCIRP.

Blood samples were obtained from deidentified healthy individuals. Five mL of blood was collected by venipuncture and placed in EDTA blood collection tubes. We isolated neutrophils from fresh blood samples by immunomagnetic cell separation using the EasySep Direct Human Neutrophil Isolation Kit (catalog 19666; Stem Cell Technologies). A total of 20 × 10^6^ neutrophils were obtained from 5 mL of blood. Neutrophils (1 × 10^6^ cells/mL) were stimulated with various doses of recombinant mouse CIRP (i.e., eCIRP), for 4 hours. The cells were then stained with anti-human FITC CD66b (clone G10F5; Biolegend), PerCP/Cy5.5 CXCR4 (clone 12G5; Biolegend), Pacific Blue CD62L (clone DREG-56, Biolegend), APC HLA-DR (clone L243; Biolegend), PE/Cy7 CD40 (clone 5C3; Biolegend), and PE CD86 (clone BU63; Biolegend) Abs, and the frequencies (percentages) of APANs were detected by flow cytometry.

### Experimental animals.

Eight-week-old C57BL/6 mice were purchased from Charles River Laboratories. CIRP^–/–^ mice were originally obtained from Jun Fujita (Kyoto University, Kyoto, Japan); TLR4^–/–^ mice were obtained from Kevin Tracey (Feinstein Institutes for Medical Research); and OT-II transgenic mice [B6.Cg-Tg(TcraTcrb)425Cbn/J] were obtained from Yong-Rui Zou (Feinstein Institutes for Medical Research). ROSA-iDTR^KI^ mice [C57BL/6-Gt(ROSA)26Sor^tm1(HBEGF)Awai^/J] and MRP8-Cre^+^ mice [B6.Cg-Tg(S100A8-cre,-EGFP)1Ilw/J] were purchased from The Jackson Laboratory. MRP8-Cre^+^ mice were crossed with ROSA-iDTR^KI^ mice to generate PMN^DTR^ mice (MRP8-Cre^+^; ROSA-iDTR^KI^). Mice were housed at approximately 23°C with 12-hour light cycles and provided standard laboratory food and water ad libitum. Only male mice were used in this study. Animals were randomly assigned to the sham, vehicle, or adoptive transfer groups.

### Animal model of polymicrobial sepsis.

Mice were anesthetized with isoflurane to a surgical plane and placed in a supine position. CLP was performed through a midline laparotomy. The abdomen was shaved and disinfected. Through a 2 cm midline incision, the cecum was exposed and then ligated with a 4-0 silk suture 1 cm proximal from the distal cecal extremity. For 20-hour CLP experiments, the cecum was punctured twice with a 22-gauge needle. A small amount of cecal content was extruded through both holes, and the ligated cecum was returned to the peritoneal cavity. The surgical wound was closed in layers. For 10-day sepsis survival studies, the cecum was punctured once with a 22-gauge needle. A small amount of cecal content was extruded. Mice were allocated to the vehicle and adoptive transfer of APANs or nAPANs groups. Adoptive transfer mice received a retro-orbital injection of either APANs or nAPANs (1 × 10^6^ cells/mouse) in 100 μL PBS immediately after abdominal closure for both the 20-hour experiments and survival studies. Vehicle groups received an equivalent volume of PBS. Sham-operated animals underwent a similar laparotomy but without CLP. After closure, the mice received a s.c. injection of 0.5 mL normal saline to overcome surgery-induced dehydration. For 20-hour experiments, animals were not injected with antibiotics; however, for survival studies, animals were given 0.5 mg/kg BW imipenem s.c. in 0.5 mL saline once at the end of CLP. All mice received a single s.c. dose of 0.1 mg/kg buprenorphine after CLP.

### Recombinant mouse CIRP administration and tissue collection.

Recombinant mouse CIRP (i.e., eCIRP) was generated and purified in-house (9). A small incision on the neck was made to expose the internal jugular vein. PBS or eCIRP at a dose of 5 mg/kg BW in 200 μL volume was delivered by jugular vein injection using a 29-gauge 0.5-inch U-100 insulin syringe (Terumo Medical Corporation). At 5 hours after eCIRP injection, mice were anesthetized, and the blood, lungs, and spleens were collected.

### APAN immunophenotyping.

250 μL whole blood obtained from sham, CLP, and PBS- or eCIRP-treated mice was taken into Falcon 15 mL conical tubes along with 5 mL red blood cell (RBC) lysis buffer (BD Biosciences). After 5 minutes of incubation at room temperature, the samples were centrifuged at 300*g* for 10 minutes. Supernatants were aspirated, and the cell pellet was washed by suspending the cells in 5 mL FACS buffer containing 2% FBS and centrifuged at 300*g* for 10 minutes. The supernatant was discarded, and the cell pellet was dissolved in 500 μL FACS buffer. Lung tissues were finely diced using a sterile surgical blade and suspended in calcium- and magnesium-free HBSS. Tissue digestion was performed in HBSS containing 100 U/mL collagenase I (Worthington Biochemical) and 20 U/mL DNase I (Worthington Biochemical) at 37°C for 30 minutes with periodic shaking. Digested tissue fragments were crushed with a 10 mL syringe plunger and passed through a 70 μm cell strainer (Corning). Lysis of RBC in lung cell suspensions was conducted using RBC lysis buffer (BD Biosciences). The isolated lung cells were counted using a microscope (Eclipse TS100; Nikon). Spleen tissues were sliced into small pieces and suspended in calcium- and magnesium-free HBSS. The cells were passed through the strainer, and the cell suspension was centrifuged at 300*g* for 5 minutes. Lysis of RBC in spleen cell suspensions was conducted using RBC lysis buffer. The numbers of isolated splenocytes were counted using a microscope. To detect mouse APANs, single-cell suspensions were stained with anti-mouse Alexa Fluor 488 Ly6G (clone 1A8; Biolegend), PerCP/Cy5.5 CXCR4 (clone L274F12; Biolegend), Pacific Blue CD62L (clone MEL-14; Biolegend), APC MHCII (clone M5/114.15.2; Biolegend), PE CD40 (clone 3/23; Biolegend), and PE/Cy7 CD86 (clone GL-1; Biolegend) Abs and assessed for the detection of APANs (Ly6G^+^CXCR4^+^CD62L^–/lo^MHCII^+^CD40^+^CD86^+^) by flow cytometry (BD LSRFortessa; BD Biosciences). Unstained cells were used to establish the flow cytometer voltage setting, and single-color positive controls were used to adjust the compensation. The acquisition was performed on 50,000 events using a BD LSR Fortessa flow cytometer (BD Biosciences), and data were analyzed with FlowJo software (Tree Star).

### Isolation and purification of BMDNs.

Mice were anesthetized by 2% isoflurane, and femurs and tibias were dissected. The bone marrow was flushed out with calcium- and magnesium-free HBSS using a 25-gauge needle into a petri dish. Suspensions of cells were filtered through a 70 μm cell strainer (Corning), and BMDNs were purified by negative selection using the EasySep mouse neutrophil enrichment kit (catalog 19762; STEMCELL). The purity of the sorted neutrophils was assessed by staining the cells with APC-Ly6G Ab (clone 1A8; Biolegend) using BD LSR Fortessa flow cytometer (BD Biosciences).

### Flow sorting of APANs.

BMDNs were stimulated with eCIRP (1 μg/mL) for 6 or 12 hours. After stimulation, cells were washed with PBS and resuspended in 1 mL FACS buffer. APANs (CD40^+^CD86^+^CXCR4^+^CD62L^–/lo^), nAPANs (CD40^–^CD86^–^CXCR4^–^), and naAPNs, (CD40^+^CD86^+^CXCR4^–^) were sorted by flow cytometry using BD FACSAria IIu (BD Biosciences).

### Droplet-based scRNA-Seq and genomic mapping.

BMDNs treated with PBS or eCIRP (1 g/mL) for 2 hours were sorted for scRNA-Seq using the 10× Genomics Chromium platform (41). Library preparation was conducted according to the recommended protocol for the NextGEM Single-Cell 3’ Library Kit v3.1 (no. 1000121; 10× Genomics). Libraries were sequenced on the Illumina NextSeq2000 sequencing platform to a mean depth of approximately 40,000 reads per cell. The Cell Ranger count pipeline (v6.0.0, 10× Genomics) was used to align FASTQs to the mouse reference genome (gex-mm10-2020-A, 10× Genomics) and produce digital gene-cell counts matrices and to perform quality control of the mapping results. Primary assessment with Cell Ranger for the PBS-treated sample reported 6,104 cells with a median of 4,624 unique molecular identifiers per cell and median of 1,284 genes per cell at 64.8% sequence saturation, with a median of 37,255 reads per cell. Primary assessment with Cell Ranger for the eCIRP-treated sample reported 5,711 cells with 4,460 unique molecular identifiers per cell and 1,250 median genes per cell at 70.0% sequence saturation, with a median of 44,261 reads per cell.

### Single-cell sequencing quantification.

DoubletFinder was used to identify doublets (42). After filtering out doublets, Seurat v4.1.3 was used to filter out cells expressing less than 200 genes and all cells with more than 5% mitochondrial genes (43). Among the PBS-treated cells, 5,597 singlets were kept, and 507 cells were removed through quality control. Among the eCIRP-treated cells, 5,200 singlets were kept, and 511 cells were removed through quality control. Gene expression normalization and cell clustering was done using the SCTransform pipeline with percentage mitochondrial reads regressed out and batch effects corrected using Harmony (44, 45). After preprocessing, “ground truth” reference-based cell classification was done using SingleR (46). Mouse immune cell gene expression profiles from the Immunological Genome Project (ImmGen) were used as reference to classify cells by main cell type (47). All nonneutrophils were filtered from the data sets by importing ImmGen cell classification labels into the Seurat object and using Seurat’s subset function to remove nonneutrophils. The neutrophils were subsequently reclustered at a high resolution of 2 to discover whether eCIRP induced an increase in the likely relatively small population of APANs. A small cluster containing these APANs was selected using expression of *Cd74* and *Cxcr4*. Neutrophils, except for APANs, were classified by using the reference transcriptomes of unstimulated and activated neutrophils (21). Noise was reduced, and sparsely represented genes were imputed using MAGIC (48).

### Neutrophil and CD4^+^ T cell cocultures.

APANs, nAPANs, and naAPNs (1 × 10^5^) were cultured with the MHC-II–restricted ovalbumin epitope (OVA 323–339 peptide, catalog RP106101; GenScript; 1 μg/mL) for 2 hours and then cocultured with CD4^+^ T cells isolated from OT-II transgenic mouse splenocytes (1 × 10^5^) for the duration specified in respective figure legends. IL-12p70 and IFN-γ in culture supernatants were quantified by ELISA (BD Biosciences).

### Adoptive transfer of APANs into neutropenic mice.

PMN^DTR^ mice were injected i.p. with 500 ng diphtheria toxoid (Sigma-Aldrich). After 24 hours, blood was collected by submandibular bleeding, and the percentage of neutrophils was quantified using an ADVIA 2120i veterinary hematology system (Siemens) and by flow cytometry. APANs and nAPANs from WT mouse BMDNs after stimulation with eCIRP (1 μg/mL for 6 hours) were sorted by flow cytometry and adoptively transferred into PMN^DTR^ mice or C57BL/6 mice by retro-orbital injection immediately after CLP. After 20 hours of CLP, plasma and lungs were harvested to analyze proinflammatory cytokines.

### Assessment of organ injury markers.

Serum levels of ALT, AST, and LDH were determined using specific colorimetric enzymatic assays (Pointe Scientific Inc.) according to the manufacturer’s instructions.

### Assessment of proinflammatory cytokines.

Cell culture supernatant and serum levels of IL-6, TNF-α, IFN-γ, and IL12p70 were analyzed by ELISA (all were purchased from BD Biosciences).

### Detection of NETs by fluorescent microscopy and ELISA.

To detect NETs using fluorescent microscopy, murine BMDNs (1 × 10^6^ cells/mL) were stimulated with IFN-γ (10 ng/mL) with or without eCIRP (1 μg/mL). At 4 hours after stimulation, the cells were stained with Sytox Green and visualized by fluorescent microscopy. We also detected NETs using our recently published ELISA protocol (49). ELISA plate for NETs assay was prepared by adding capture Ab (anti-mouse neutrophil elastase, Biotin, catalog ab 79962; Abcam; 1:2,000 dilution in sterile PBS) to 96-well high-binding capacity streptavidin-coated ELISA microplates (Roche Cell Death ELISA Kit plates, catalog 11774425001; Millipore Sigma) and incubating the plates overnight at 4°C to allow binding of capture Abs. Afterward, the plate was washed to remove unbound Ab and blocked with 5% BSA for 2 hours. NETs collected from APANs, nAPANs, and naAPNs were added to the plate and incubated overnight at 4°C to allow the protein component of NETs to bind to capture Abs. We then washed and added detection Ab to ELISA plates (anti-DNA-POD Ab, Cell Death Detection Kit, catalog 11544675001, Roche Diagnostics GmbH; 1:500, mouse, 50 μL/well) and incubated for 1 hour in the dark at room temperature. Next, we added TMB peroxidase substrate to each well for 30 minutes (50 μL/well), then stopped the reaction by adding 1 M HCl to each well (50 μL/well), and read absorbance at 450 nm using a microplate photometer. We analyzed the results either as absorbance values (relative quantitation) or a percentage of the NETs standards. The NET standard consisted of pooled DNase-digested NETs released by eCIRP-stimulated neutrophils. We added biotinylated primary Ab to streptavidin-coated ELISA plates because the biotin-streptavidin coating system is more efficient than coating buffer (50). In this ELISA method, we measured extracellular DNA that contained neutrophil elastase to determine NETs with high specificity, because NETs are the only form of cell-free DNA known to contain neutrophil elastase.

### Real-time qPCR.

mRNA was extracted from lung tissues with TRIzol reagent (Invitrogen). An equal amount of mRNA was reverse transcribed into cDNA using the reverse transcriptase enzyme (Applied Biosystems). The qPCR was performed from diluted cDNA templates with forward and reverse primers (Supplemental Table 3) and SYBR Green PCR Master Mix (Applied Biosystems) using Applied Biosystems 7300 real-time PCR machine. Mouse β-actin served as an internal control gene for normalization. Relative expression of mRNA was represented as fold change compared with the sham group.

### Pulmonary edema and histologic examinations.

To assess pulmonary edema, we measured the lung wet weight–to-BW ratio 20 hours after CLP (51). Directly prior to euthanasia mouse BW was measured. We then performed a postmortem laparotomy and thoracotomy. The left bronchus and pulmonary artery were clamped, and we resected the left lung at the hilum to obtain its wet weight. With the left bronchus and pulmonary artery still clamped, we transected the aorta and injected 2.5 mL of 1× PBS with 10 U/mL heparin into the right ventricle of the heart at a rate of approximately 300 μL/s to perfuse the right lung. Then, we inflated the right lung by injecting 10% formalin into the trachea at a rate of approximately 200 μL/s until it was fully inflated, as confirmed by backflow of formalin out of the trachea. Lung tissues were fixed in 10% formalin before being embedded in paraffin. Blocks were cut into 5 μm sections and stained with H&E. Slides were evaluated under light microscopy to assess the degree of lung injury using the American Thoracic Society scoring system (51). Scores ranged from 0 to 1 and were based on proteinaceous debris in the airspaces, the degree of septal thickening, and neutrophil infiltration in the alveolar and interstitial spaces. The average score per field was calculated at ×400 magnification.

### Statistics.

Data are expressed as mean ± SEM and were compared using unpaired 2-tailed Student’s *t* test for 2 groups or 1-way ANOVA followed by the Student-Newman-Keuls (SNK) post hoc analysis for multiple groups. Survival rates were analyzed by Kaplan-Meier and compared using the log-rank test. Differences were considered statistically significant at *P* ≤ 0.05. Data analysis was carried out using GraphPad Prism graphing and statistical software. All samples included in this study were used for statistical analysis.

### Study approval.

The human participant protocol (IRB protocol 22-0458) for collecting blood samples from deidentified patients and healthy individuals was approved by the Northwell Health Institutional Review Board, and written informed consent was obtained from the participants. All experiments involving mice were conducted in accordance with the NIH guidelines for experimental animals, and the animal protocol was approved by Feinstein Institutes for Medical Research’s Institutional Animal Care and Use Committee.

### Data and code availability.

The scRNA-Seq data for this study can be accessed at the NCBI Gene Expression Omnibus (GEO GSE228482). All scRNA-Seq data analyses were conducted using established protocols with the previously described sctransform (SCT) Seurat pipeline in conjunction with Harmony (44, 45).

## Author contributions

HJ and MA designed the experiments. HJ, AM, and MK conducted the experiments and acquired the data. HJ, MA, AM, MB, and PW analyzed the data. MK collected the patients’ samples and assisted in the analysis and interpretation of APAN findings in human blood. AJS, RPA, MA, and AM analyzed scRNA-Seq data. MA, HJ, and AM wrote the manuscript. MB and PW reviewed and edited the manuscript. PW conceived the idea. MA, MB, and PW supervised the project.

## Supplementary Material

Supplemental data

## Version 1 07/17/2023

Electronic publication
