# Supplementary material for: Antigen-presenting aged neutrophils induce CD4+ T cells to exacerbate inflammation in sepsis
Source: J Clin Invest. 2023 Jul 17;133(14):e164585. doi: 10.1172/JCI164585 (PMC10348768; doi:10.1172/JCI164585)
Supplement: Supplemental data [file jci-133-164585-s057.pdf]

Supplemental Figure 1

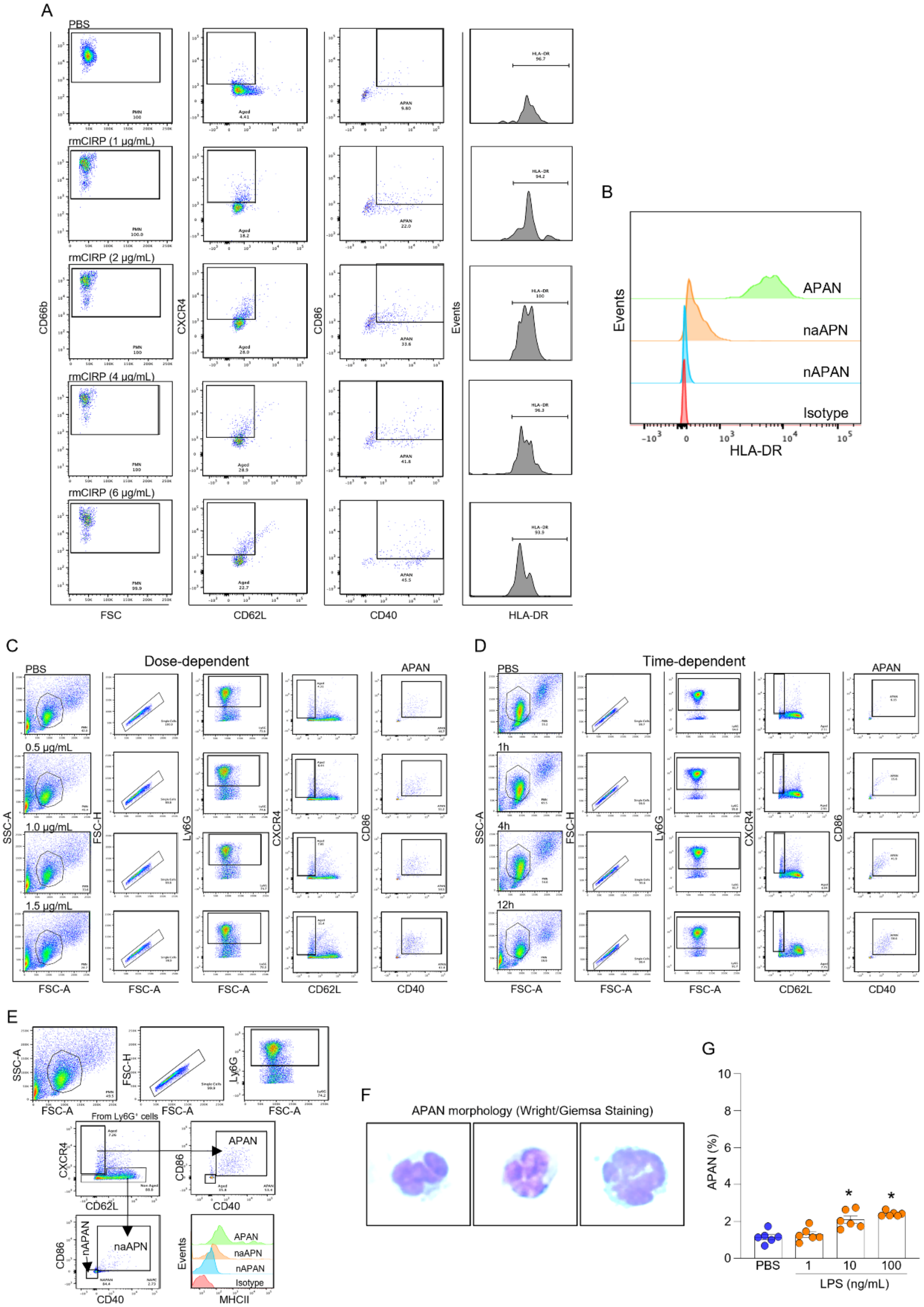

Supplemental Figure 1: Flow cytometry gating strategy for the detection of APANs and the effect of LPS on APAN formation. (A) Human peripheral blood neutrophils ($1{\times}10^6$/mL)

obtained from healthy volunteers were stimulated with various doses of eCIRP for 4 h. The cells were then stained with anti-CD66b, CXCR4, CD62L, CD86, CD40, HLA-DR, and isotype Ab, followed by the detection of APANs by flow cytometry. Representative images of the gating strategy are shown. **(B)** HLA-DR expression in eCIRP-treated human blood neutrophils was assessed in various neutrophil populations (APANs, naAPNs, and nAPANs) by flow cytometry as presented in the histogram. BMDNs (1×10^6) were treated with eCIRP at different **(C)** doses and **(D)** time-points and then the cells were stained with anti-Ly6G, CD40, CD86, CXCR4, and CD62L and subjected to flow cytometry. **(E)** MHC-II expression was assessed in various cell population by flow cytometry as presented in the histogram. **(F)** Morphology of APANs. APANs were sorted from splenic cells of CLP mice, placed on a slide glass, and stained with Wright-Giemsa solution (Sigma-Aldrich). Samples were observed with microscope at 1000× magnification. n=3 APAN cells are shown as representative images. **(G)** Effect of LPS on APAN formation. Mouse BMDNs (1×10^6/mL) were treated with LPS at various doses for 12 h. The frequency of APANs was then assessed by flow cytometry. Experiments were performed at least 3 times, and all data were analyzed. Data are expressed as mean ± SEM and compared by one-way ANOVA and SNK test. n=6/group. *$p<0.05$ vs. PBS. BMDNs, bone marrow-derived neutrophils; APAN, antigen-presenting aged neutrophil.

Supplemental Figure 2

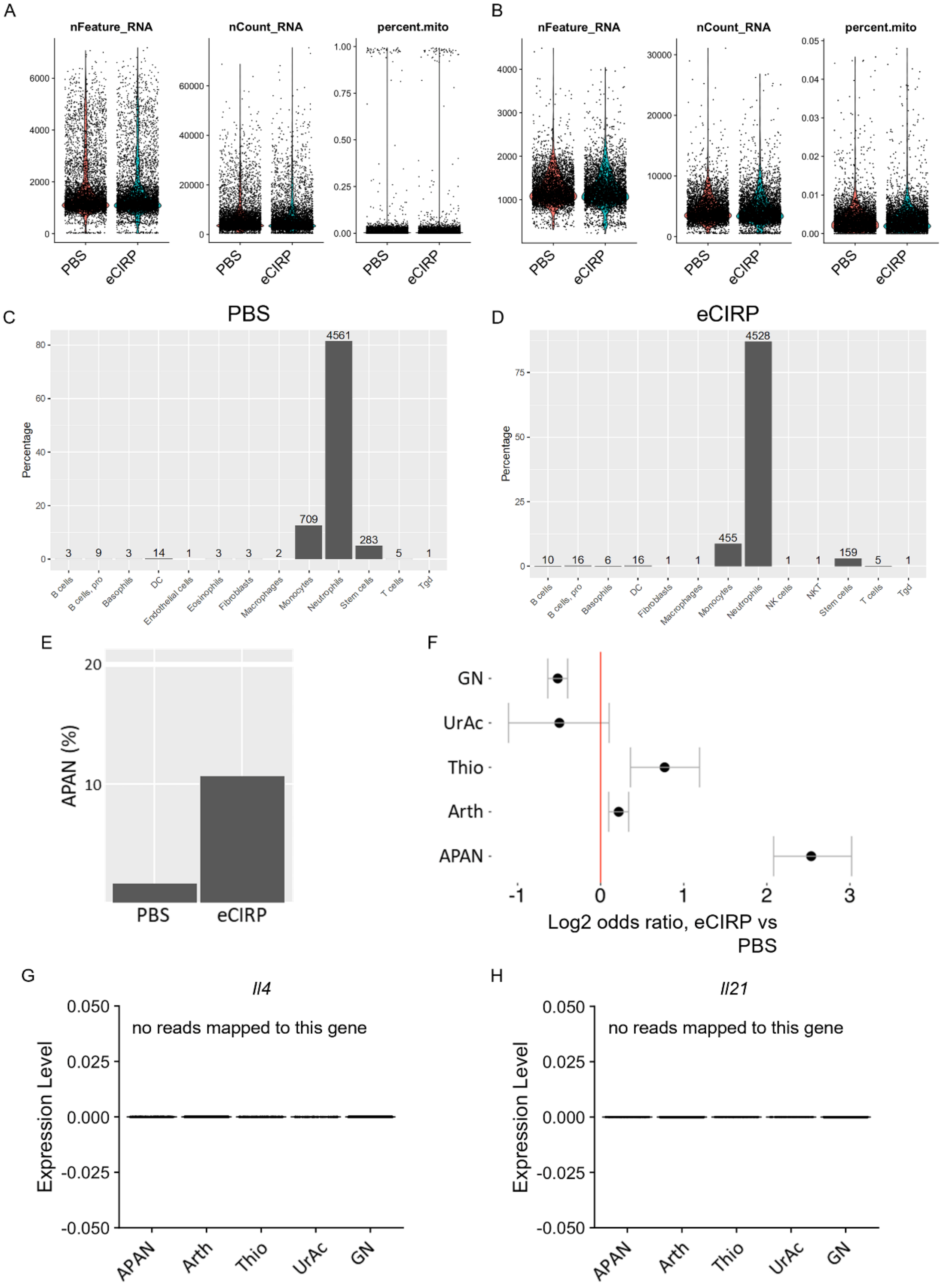

Supplemental Figure 2: Quality control data of single-cell RNA sequencing. (A) Plots showing the number of genes detected in each cell (nFeature_RNA), the number of molecules

detected in each cell (nCount_RNA), and percent mitochondrial genes (percent.mito) prior to and **(B)** after filtering. **(C)** Plots showing the percentage and counts of each cell type in the PBS group and **(D)** in the eCIRP-stimulated group. **(E)** Increase in the percentage of APANs after stimulation with eCIRP. **(F)** eCIRP-induced changes in the percentage of cells matching ImmGen mouse neutrophil transcriptomes. **(G)** Graphs showing differential expression of *Il4* and **(H)** *Il21* across different neutrophil transcriptomes. No reads were detected to these genes. BMDN, bone marrow-derived neutrophils; APANs, antigen-presenting aged neutrophils; eCIRP, extracellular cold-inducible RNA-binding protein; Immunological Genome Project, ImmGen; unstimulated circulating neutrophils, GN; arthritic mouse neutrophils, Arth; thioglycolate-simulated peritoneal neutrophils, Thio; uric acid-simulated peritoneal neutrophils, UrAc. APAN, antigen-presenting aged neutrophil; eCIRP, extracellular cold-inducible RNA-binding protein.

Supplemental Figure 3

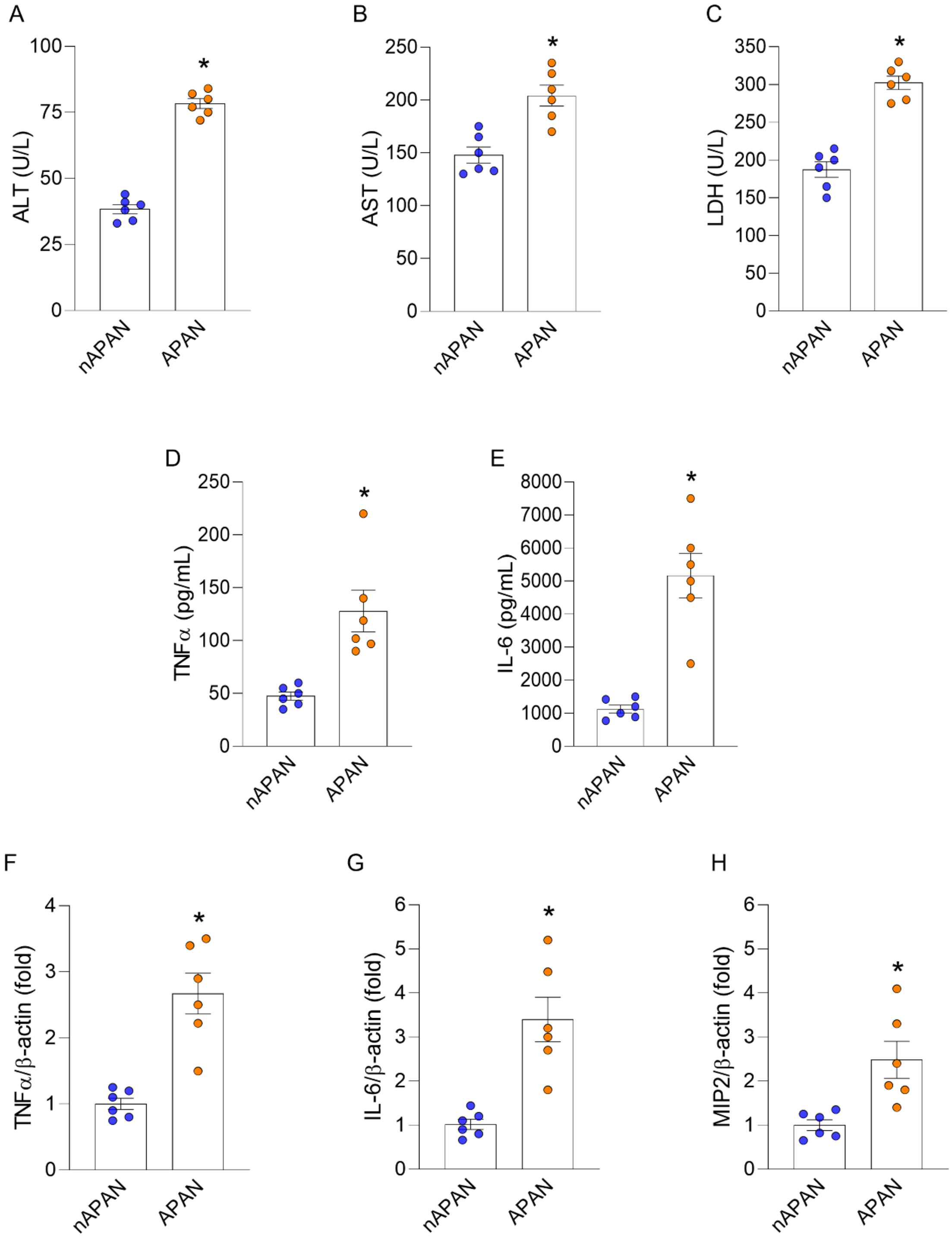

Supplemental Figure 3: BMDNs were isolated from WT mice and stimulated with eCIRP for 6 h and after that the cells were stained with anti-Ly6G, CXCR4, CD62L, CD40, and CD86 Ab and then APANs and nAPANs were sorted by FACS. A total of 1×10^6 each of APANs, and

nAPANs were adoptively transferred into neutropenic mice (PMN^{DTR} mice) via retro-orbital injection at the time of CLP. At 20 h later, blood and lungs were collected to assess several parameters. Serum **(A)** ALT, **(B)** AST, and **(C)** LDH were determined using specific colorimetric enzymatic assays. Serum **(D)** TNFα, and **(E)** IL-6 levels were measured by ELISA. Lung mRNA levels of **(F)** TNFα, **(G)** IL-6, and **(H)** MIP2 were measured by real-time PCR. Data are expressed as means ± SE (n=6 mice/group) and compared by one-way ANOVA and SNK method (*$p<0.05$ vs. CLP+nAPAN-injected mice). APAN, antigen-presenting aged neutrophil; nAPAN, non-antigen-presenting aged neutrophil; CLP, cecal ligation puncture; ALT, alanine aminotransferase; AST, aspartate amino transferase; LDH, lactate dehydrogenase; IL, interleukin; TNF, tumor necrosis factor; MIP2, macrophage chemoattractant protein 2.

Supplemental Table 1: Clinical parameters of patients with sepsis

Patient	Age	Sex	SOFA*	APACHE-II*	Hours#	Initiating Clinical Events
1	65	F	7	26	21	Iatrogenic injury of transverse colon with associated feculent peritonitis
2	71	F	5	14	20	Perforated ischemic bowel secondary to parastomal hernia with associated feculent peritonitis, incidentally COVID-19^{+}
3	66	M	6	12	22	Perforated appendicitis
4	69	F	8	19	19	Deep surgical site infection after incarcerated hernia repair

*SOFA and APACHE-II scores upon presentation; #Time from identification of sepsis to blood sample collection, hours

Supplemental Table 2: WBC and neutrophil counts in WT and PMN^{DTR} mice (MRP8-Cre^{+} × ROSA-$iDTR^{KI}$)

Mice strains	WBCs (x10^3 cells/μL)	Neutrophils (x10^3 cells/μL)
WT	7.58 ± 3.79	2.93 ± 1.46
PMN^{DTR} mice (MRP8-Cre^{+} × ROSA-$iDTR^{KI}$)	4.12 ± 2.06*	0.76 ± 0.38*

N=3/5 mice/group; Student's T-Test; *$p<0.05$ vs WT mice

Supplemental Table 3: Mouse primer sequences

Gene	Forward (5'-3')	Reverse (5'-3')
TNFα	AGACCCTCACACTCAGATCATCTTC	TTGCTACGACGTGGGCTACA
IL-6	CCGGAGAGGAGACTTCACAG	CAGAATTGCCATTGCACAAC
KC	GCTGGGATTCACCTCAAGAA	ACAGGTGCCATCAGAGCAGT
MIP2	CCCTGGTTCAGAAAATCATCCA	GCTCCTCCTTTCCAGGTCAGT
β-actin	CGTGAAAAGATGACCCAGATCA	TGGTACGACCAGAGGCATACAG
